# Epigenetic Mechanisms Underlying the Link between Non-Alcoholic Fatty Liver Diseases and Nutrition

**DOI:** 10.3390/nu6083303

**Published:** 2014-08-21

**Authors:** Joo Ho Lee, Simonetta Friso, Sang-Woon Choi

**Affiliations:** 1 Department of Gastroenterology, Bundang CHA Hospital, CHA University School of Medicine, Seoul 463-712, Korea; E-Mail: piolee2000@naver.com; 2 Department of Medicine, University of Verona School of Medicine, Verona 37134, Italy; E-Mail: simonetta.friso@gmail.com; 3 Clinical Genomic Center, Chaum Life Center, CHA University School of Medicine, Seoul 135-948, Korea

**Keywords:** epigenetics, non-alcoholic fatty liver disease (NAFLD), non-alcoholic steatohepatitis (NASH), one-carbon metabolism, nutrition

## Abstract

Non-alcoholic fatty liver disease (NAFLD) is defined as a pathologic accumulation of fat in the form of triglycerides (TG) in the liver (steatosis) that is not caused by alcohol. A subgroup of NAFLD patients shows liver cell injury and inflammation coupled with the excessive fat accumulation (steatohepatitis), which is referred to as non-alcoholic steatohepatitis (NASH). Patients with NASH may develop cirrhosis and hepatocellular carcinoma (HCC). NAFLD shares the key features of metabolic syndrome including obesity, hyperlipidemia, hypertension, and insulin resistance. The pathogenesis of NAFLD is multi-factorial, however the oxidative stress seems to plays a major role in the development and progression of the disease. The emerging field of epigenetics provides a new perspective on the pathogenesis of NAFLD. Epigenetics is an inheritable but reversible phenomenon that affects gene expression without altering the DNA sequence and refers to DNA methylation, histone modifications and microRNAs. Epigenetic manipulation through metabolic pathways such as one-carbon metabolism has been proposed as a promising approach to retard the progression of NAFLD. Investigating the epigenetic modifiers in NAFLD may also lead to the development of preventive or therapeutic strategies for NASH-associated complications.

## 1. Introduction

Non-alcoholic fatty liver disease (NAFLD), ranging from simple steatosis through steatohepatitis to, ultimately, cirrhosis, is characterized by an abnormal accumulation of triglycerides (TG) in the liver without alcohol consumption [1]. NAFLD is often associated with the most common clinical features of metabolic syndrome, such as central obesity, type 2 diabetes mellitus, dyslipidemia and arterial hypertension [2]. It is generally considered a benign condition, affecting up to 60%–70% of diabetic and obese patients [3]. About 25% of patients affected by NAFLD progress to non-alcoholic steatohepatitis (NASH), which is characterized by inflammation, hepatocellular ballooning degeneration, fibrosis and liver cell injury [4,5,6]. A pathologic study using human liver biopsy specimens revealed that cirrhosis can develop in about 25% of patients with NASH [7]. Most individuals with NAFLD remain asymptomatic, while patients with NASH may develop to cryptogenic liver cirrhosis, end-stage liver disease or even hepatocellular carcinoma (HCC) [8,9].

The prevalence of NAFLD in the general population of Western countries ranges from 20% to 30% [10,11]. Population based screening has estimated that at least 25% of the general population in the United States (US) has NAFLD [12]. Furthermore, the incidence ratio of NASH has increased in recent years of up to 8% of US adults, which reflects a substantial proportion of individuals at risk of NAFLD-related morbidity [4,12]. The incidence of obesity has rapidly increased in Korea, resulting in a high prevalence of NAFLD in the Korean population, ranging from 20% to 25% [13,14,15]. In many other Asian countries, the incidence and prevalence of obesity-related NAFLD are also increasing due to the ongoing socioeconomic transition and shift toward Westernized diet [10,16,17]. Since obesity is strongly associated with NAFLD [18,19], the prevention of obesity is now a major public issue in Asian countries similar to Western countries [2,5,20]. Until recently, pharmacologic treatment of NAFLD-associated obesity remained uncertain [21]. Thus, a reduction in total energy intake and body weight with appropriate dietary modifications and exercise has been the cornerstone of NAFLD treatment [14,22,23,24].

The pathogenesis of NAFLD is not yet entirely understood, but it seems that it is multi-factorial [1,25]. Liver fat accumulation is mainly induced by insulin resistance and increased free fatty acids, and NASH is developed by oxidative stress, mitochondrial dysfunction, and cytokine interplay [26,27,28]. Interestingly, genetic and environmental factors such as exercise and diet interact to define the NAFLD phenotype and determine its progression [29,30]. A large proportion of the population is at the greater risk of NAFLD owing to the high prevalence of obesity and insulin resistance, but only a limited number of individuals affected by those conditions develops NASH and its associated morbidity [31], suggesting many other factors are involved in the development of this disease. Among them is the individual genetic susceptibility to NASH [30].

Because liver is central for the whole-body metabolism, NAFLD leads to changes in cell transcriptional status that may cause a perturbation in energy metabolism, contributing to the development and progression of many chronic diseases, including atherosclerosis and type 2 diabetes mellitus [32]. The dysregulation of energy metabolism has been thought to be conveyed by epigenetic mechanisms, thereby changing the expression of critical genes [2,33]. Indeed the emerging field of “epigenetics” provides new perspectives on the pathogenesis of chronic liver diseases, including NAFLD [34]. Comprehensive investigations of epigenetic marks that predispose an individual to NAFLD may lead to the development of novel biomarkers for early diagnosis of NAFLD and may allow early preventive or therapeutic strategies for the people at the high risk for NASH and NASH associated HCC [8,10]. This review is focused on the currently available knowledge on epigenetic modifiers that influence the development and progression of NAFLD.

## 2. Epigenetic Mechanism Underlying Disease Development

Epigenetics describes reversible changes in gene expression that can be inherited through mitosis and/or meiosis and finally affects the phenotype by allowing the fine-tuning of gene transcription without altering the primary DNA sequence [35]. It is known that the gene expression can be regulated by any of the following control steps: (a) chromatin structure; (b) initiation of transcription; (c) processing of the transcript; (d) transport to the cytoplasm; (e) translation of mRNA; and (f) the stability of protein activity [36]. The epigenetic phenomena regulate the chromatin structure modifications and the initiation of transcription in a manner that alters the availability of genes to transcription factors required for their expression [37].

At present, four mechanisms responsible for mediating epigenetic effects are: (a) DNA methylation (occurring at the 5′-position of cytosine residues within CpG dinucleotides); (b) histone modifications (acetylation, methylation, phosphorylation, ubiquitination, ribosylation, biotinylation and sumoylation of histone tails); (c) chromatin remodeling; and (d) possibly RNA-based mechanisms such as microRNA (miR) [3]. The most studied epigenetic marks are DNA methylation and posttranslational modifications of histones [38]. The enzymes involved in these reactions include DNA methyltransferases (DNMTs), histone methyltransferases (HMTs), histone demethylases (HDMs), histone acetyltransferases (HATs), and histone deacetylases (HDACs) [39,40,41].

During the past decade, cancer has been the most extensively studied field in epigenetics [38,42]. In fact epigenetic dysregulation of critical gene expression has been known to initiate cancer development and contribute to cancer progression [8,43]. As epigenetic modifications are reversible, the new therapeutic strategies to modulate epigenetic aberrancies are now being extensively investigated [43,44]. In contrast, the role of epigenetics in the pathogenesis of NAFLD has not been completely elucidated until now. The epigenetic studies of NAFLD and associated metabolic syndrome still remain in its infancy. Increasing our understanding of epigenetics and disease-associated epigenetic patterns should provide important future directions to develop novel strategies to cope with the growing worldwide epidemic of obesity, metabolic syndrome, and fatty liver diseases [10,37].

## 3. Potential Role of Epigenetics in NAFLD

Since liver is a central organ in lipogenesis, an abnormal accumulation of TG can impair hepatic functions and lead to steatohepatitis, cirrhosis and possibly HCC [32]. There is some evidence showing that the dysregulation of hepatic function is conveyed by epigenetic mechanisms in NAFLD [2]. Targeted epigenetic manipulation of certain metabolic or stress-response pathways such as one-carbon metabolism or nuclear transcription factor-κB (NF-κB) signaling has been highlighted in clarifying the pathways that regulate disease progression in NAFLD [5,10]. In fact, epigenetic phenomena are modulated by environmental stimuli, such as stress and nutritional status [45,46]. Recently, several examples of dynamic changes in epigenetic marks by nutritional interventions have been reported, including the use of the DNA methylation profile as a prognostic biomarker of diet response [47,48]. The liver is responsible for many biological methylation reactions including the methylation of DNA and histones [2]. The interplay between chromatin-modifying enzymes and hepatic transcription factors may induce the epigenomic reprogramming that may provide a milieu leading to malignant conversion of hepatocytes [8,49]. The candidate epigenetic mechanisms for NAFLD are summarized at Table 1.

**Table 1:** Epigenetic mechanism for non-alcoholic fatty liver disease.

Mechanism	Subject	Study Result	References
DNA Methylation	Mouse	Hepatic epigenetic phenotype predetermines individual susceptibility to hepatic steatosis.	Pogribny* et al.* [50]
	Human	Hepatic methylation and transcriptional activity of the*MT-ND6* are associated with the histological severity of NAFLD.	Pirola* et al.* [51]
	Mouse	Puffs from dams fed a high fat diet display characteristics of NAFLD phenotype and associated changes in gene expression and DNA methylation.	Dudley* et al.* [52]
	Mouse	Coupling global methylation and gene expression profiles reveal key pathophysiologic events in liver injury.	Tryndyak* et al.* [53]
	Human	Epigenetic regulation of insulin resistance in NAFLD: Impact of liver methylation of the PPARγ coactivator 1alpha promoter.	Sookoian* et al.* [54]
	Human	Altered methylation of genes that regulate processes such as steatohepatitis, fibrosis, and carcinogenesis indicate the role of DNA methylation in progression of NAFLD.	Murphy* et al.* [55]
Histone Modifications	Mouse	Role of the histone H3 lysine 4 methyltransferase, SET7/9, in the regulation of NF-κB-dependent inflammatory genes. Relevance to diabetes and inflammation.	Li* et al.* [56]
	Mouse	Inhibition of hepatic p300 activity may be beneficial for treating hepatic steatosis in obesity and identify specific p300 inhibitors as potential targets for therapy.	Bricambert* et al.* [57]
	Mouse	A circadian rhythm orchestrated by histone deacetylase 3 controls hepatic lipid metabolism.	Feng* et al.* [58]
	Mouse	When challenged with a high-fat diet, liver-specific *Sirt1* knockout mice develop hepatic steatosis and inflammation.	Purushotham* et al.* [59]
	Mouse	Loss of *Sirt3* and dysregulation of mitochondrial protein acetylation contribute to the metabolic syndrome and NASH development.	Hirschey* et al.* [60]
Mechanism	Subject	Study Result	References
MicroRNA	Human	Nonalcoholic steatohepatitis is associated with altered hepatic microRNA expression.	Cheung* et al.* [61]
	Mouse	Deletion of mouse miR-122 resulted in hepatosteatosis, hepatitis, and the development of tumors resembling HCC.	Hsu* et al.* [62]
	Mouse	The up-regulation of miR-335 is associated with lipid metabolism in liver and white adipose tissue of obese mice.	Nakanishi* et al.* [63]
	Mouse	Difference in the expression of hepatic microRNAs (miR-29c, miR-34a, miR-155, and miR-200b) is associated with strain-specific susceptibility to dietary nonalcoholic steatohepatitis in mice.	Pogribny* et al.* [64]
One-carbon metabolism	Mouse	Absence of *Matla* resulted in a liver that is more susceptible to injury, expresses markers of an acute phase response, and displays increased proliferation.	Lu* et al.* [65]
	Mouse	A critical role for *S*-adenosylmethionine in maintaining normal hepatic function and tumorigenesis of the liver.	Martinez-Chantar* et al.* [66]
	Mouse	Loss of the glycine *N*-methyltransferase gene leads to steatosis and hepatocellular carcinoma in mice.	Martinez-Chantar* et al.* [67]
	Mouse	Hepatic PC synthesis is a key player in maintaining serum VLDL and HDL, and also important in hepatic HDL formation.	Jacobs* et al.* [68]
	Human	l-Carnitine supplementation to diet is useful for reducing TNF-α and CRP, and for improving liver function, serum glucose level, lipid profile and histological manifestations of NASH.	Malaguarnera* et al.* [69]

Abbreviations: CRP, C-reactive protein; HCC, hepatocellular carcinoma; NAFLD, non-alcoholic fatty liver disease; NASH, non-alcoholic steatohepatitis; MT-ND6, mitochondrially encoded NADH dehydrogenase 6; PC, phosphatidylcholine; PPARγ, peroxisome proliferator-activated receptor gamma; MAT, methionine adenosyltransferase; miR, microRNA; TNF, tumor necrosis factor.

### 3.1. DNA Methylation in NAFLD

The earliest discovery of epigenetic gene-silencing was DNA methylation [70]. DNA methylation refers to the addition of a methyl group on cytosine with guanine as the next nucleotide, known as CpG sites [71,72]. The clustering of CpG dinucleotides (usually referred as CpG island) is commonly present with higher frequency at the promoter regions of the genes than other sites [35]. Hypermethylation of CpG islands is generally associated with gene repression, while hypomethylation of promoter region may induce gene activation. Global DNA hypomethylation is known to influence the genome stability [10]. Such characteristics are connected to malignant transformation [73,74]. Enzymes that catalyze DNA methylation utilize *S*-adenosylmethionine (SAM) generated by one-carbon metabolism [75]. The DNA methylation patterns are known to be maintained by the functions of DNMTs. In humans, three functional DNMT isoforms have been identified: DNMT1, DNMT3A, and DNMT3B. DNMT1 is responsible for the maintenance of DNA methylation, while DNMT3A and DNMT3B catalyze *de novo* DNA methylation to establish new DNA methylation [76].

In a mouse model the development of hepatic steatosis was accompanied by changes in Dnmt1 and Dnmt3a expression in the liver [50]. Hepatic DNMT1 level was significantly increased in patients with NASH [51]. Pronounced global DNA hypomethylation and aberrant DNA methylation at specific gene promoter regions were found in steatosis and NASH developed from mice fed with lipogenic diet [50]. In an animal study, promoter DNA methylation of the glucokinase gene (*Gck*) decreased the expression of protein level and kinase activity in the rat liver, thereby increasing the risk of hyperglycemia and fatty liver [77]. Rat offsprings exposed prenatally to high fat diet also had the NAFLD phenotype, as well as increased expression of hepatic cell cycle inhibitor *Cdkn1a*, which is known to be hypomethylated at specific DNA sites during the perinatal periods [52]. This might be suggestive of early hepatic dysfunction in puffs from dams fed high fat diet, which may be associated with the process of demethylation and remethylation during the development of germ cells, referred to as “epigenetic fetal programming” [3,78].

Comprehensive genome-wide methylation analysis found extensive DNA methylation changes in more than a hundred of genes associated with lipid and glucose metabolisms, DNA damage and repair, fibrosis and liver tissue remodeling [53]. The mitochondrial gene NADH dehydrogenase 6 gene (MT-ND6) was transcriptionally silenced by promoter hypermethylation, which was significantly associated with the histological severity of NAFLD [51]. The hepatic promoter methylation of the peroxisome proliferative activated receptor (PPAR)-gamma coactivator one alpha (*PGC1-α*) gene, a key transcriptional regulator of mitochondrial fatty acid oxidation, not only correlated with the status of peripheral insulin resistance, but also associated with the fasting insulin levels of NAFLD patients [54]. In a whole-genome promoter DNA methylation analysis of skeletal muscle, *PGC1-α* hypermethylation was found in diabetic subjects [79]. Methylation levels negatively correlated with the expression of *PGC1-α* mRNA and mitochondrial density. Interestingly, non-CpG methylation of *PGC1-α* was increased by tumor necrosis factor (TNF)-α or free fatty acids, which can be elevated in the metabolic syndrome and NAFLD. Selective silencing of the *DNMT3B*, excluding *DNMT1* and *DNMT3A*, prevented TNF-α induced non-CpG methylation of *PGC1-α* and consequently increased *PGC1-α* mRNA. Non-CpG site methylation is quite rare in human DNA compared with CpG methylation, but it is also known to affect gene expression.

Growing evidence indicates that hepatic DNA methylation and insulin resistance in NAFLD patients are critical factors for the conversion from simple steatosis to severe fibrotic NASH [8]. A recent methylome and transcriptome study found that differentially methylated genes may distinguish patients with advanced NASH from simple steatosis [55]. Such integrated omics studies have increasingly revealed the critical role of DNA methylation in the progression of NAFLD (Table 1).

### 3.2. Histone Modifications in NAFLD

In the mid-1990s, histone modifications were discovered as an epigenetic determinant of chromatin structure and gene expression [80,81]. Among them is histone acetylation, the acetylation of lysine residues at the N terminus of histone tails catalyzed by HAT [82]. Histone acetylation is usually associated with the activation of gene transcription. On the other hand, histone deacetylation is catalyzed by HDAC and involved in gene repression [83]. Indeed, altered expression and activity of certain histone acetylation modifying enzymes have been reported to influence gene expression in NAFLD, leading to altered hepatic metabolism and cellular transformation [8] (Table 1). The understandings of this epigenetic mechanism underlying NAFLD may provide new perspectives in the identification of novel epigenetic targets for the management of NAFLD [49,84].

Aberrant histone modifications contribute to the development of insulin resistance and consequently to fatty liver disease [85]. Histone acetylation is dependent on the enzymatic conversion of glucose-derived citrate to acetyl-CoA, linking nutrient metabolism to epigenetic control [86]. The imbalance between HAT and HDAC has been reported to influence the histone acetylation status and phenotypic gene expression in NAFLD, resulting in the perturbation of hepatic metabolism and liver injury [8]. Among the HAT family members, the transcriptional coactivator p300 is an important component of the transcriptional regulator involved in the NF-κB dependent inflammatory pathways [87]. Poor glycemic control increases NF-κB activity and the expression of genes encoding inflammatory cytokines via interplay between NF-κB and HAT, e.g., p300 [3,88]. The methyltransferase SET7/9, which targets lysine residue 4 of histone H3 (H3K4), affects the recruitment of NF-κB p65 to gene promoters and subsequently promotes the expression of NF-κB induced inflammatory cytokines [56]. The transcription factor carbohydrate-responsive element-binding protein (CBP) has also emerged as a major player in the development of hepatic steatosis and type 2 diabetes mellitus [89]. The glucose-activated p300 also increased the CBP transcription activity. Thus, p300 contributes to the development of NAFLD through enhanced glycolytic and lipogenic gene activation via histone and non-histone protein acetylation [8,57].

Several HDACs are known to play a pivotal role in the pathogenesis of NAFLD. Defects in the regulation of circadian clock genes by HDAC3 may lead to abnormal lipid metabolism in the liver [58]. Misalignment between the circadian rhythms of HDAC3 recruitment to target metabolic genes with behavioral patterns alters lipid metabolism causing NAFLD [90]. Liver-specific deletion of HDAC3 causes both advanced fibrotic NAFLD and HCC [91]. Additionally, NAD-dependent sirtuins (class III HDAC, SIRT), which target both histones and non-histone proteins, mediate adaptive responses to metabolic stress and regulate adipogenesis and insulin secretion [92]. SIRT1 inhibits NF-κB activity to reduce inflammatory response and modulates other cytokines involved in lipid metabolism [8,93]. Thus, liver-specific deletion of SIRT1 had increased fatty liver disease and obesity induced inflammation, while SIRT1 over-expression showed protective effects against steatohepatitis and insulin resistance [94,59]. SIRT1 improves insulin sensitivity under the insulin-resistant conditions by repressing protein tyrosine phosphatase 1B (PTP1B), a negative regulator of insulin signaling, and is recruited to telomeric repeats to enhance genomic stability [95,96,97]. In a high-fat diet murine model exposed to chemical carcinogen, over-expression of SIRT1 effectively reduced the development of HCC. This tumor suppressive effect might be attributed to the dual effects that ameliorate DNA damages elicited by both chemical carcinogen and high fat diet [98].

One of the molecular targets of SIRT1 is macroH2A1, a variant of histone H2A, which is involved in hepatic lipid metabolism and is present in two alternative spliced isoforms, macroH2A1.1 and macroH2A1.2 [99]. Immunopositivity for both macroH2A1 isoforms were markedly upregulated in HCC, whereas macroH2A1.2 was specifically upregulated in steatosis. A recent study by Pazienza* et al.* [100] showed that over-expression of SIRT1-metabolite binding macroH2A1.1 can protect hepatocytes against lipid accumulation. Otherwise, the SIRT3 localizes mainly in the mitochondria, and it is required for maintenance of mitochondrial integrity upon oxidative stress [101]. SIRT3 deficient mice have been reported to display NASH and perturbation of the SIRT3 activity in mice was associated with the abnormalities similar to metabolic syndrome and NAFLD [60]. Both the SIRT1 and SIRT3 are very important in the homeostatic balance of redox status, epigenetic alteration and lipid metabolism in the hepatocytes. These metabolic cascades of histone deacetylase activity are intertwined in NAFLD pathogenesis.

Epigenetic mechanisms of nuclear chromatin remodeling including post-translational modifications of histones, and incorporation of histone variants into the chromatin are also increasingly recognized as crucial factors in the pathophysiology of NAFLD [78]. Likewise, the histone amino-terminal modifications can generate dynamic transitions between transcriptionally active and silent chromatin states [102]. Thus, the combinatorial nature of histone amino-terminal modifications reveals a “histone code” that considerably extends the potential information of the genetic code and plays an essential role in gene expression [103].

### 3.3. microRNAs (miRs) in NAFLD

In the early 2000s, non-coding miRs were identified and their epigenetic properties were characterized [104]. Indeed, miRs are the most extensively investigated epigenetic mechanism in NAFLD relative to the other epigenetic machineries [10]. miRs are small, naturally occurring single-stranded RNA (18–25 nucleotides in length) regulating mRNA degradation or protein translation, ultimately affecting the phenotypic expression of target genes [104,105]. miRs typically regulate transcription in either a positive or a negative manner through the inhibition of translation or the increased degradation of target mRNAs [106]. miRs function in the context of the RNA-induced silencing complex (RISC), whereby the miR directs RISC to target mRNAs via complementary base pairing.

Aberrant expression of miRs has been implicated in obesity, insulin resistance, type 2 diabetes mellitus, and fatty liver disease [107,108]. Recently, it has been shown that about 100 miRs are differentially expressed in human NASH [10]. These miRs have diverse functions involved in the pathogenesis of steatohepatitis, including the regulation of lipid and glucose metabolisms, oxidative stress, cellular differentiation, inflammation, and cell survival pathways [109,61] (Table 1).

The miR-122, a highly abundant miR in the liver, has been known to perform a major role in the pathogenesis of liver diseases including both the metabolic and viral hepatitis [61,110,111,112,113]. Accounting for nearly 70% of all miRs in the liver, miR-122 is significantly under-expressed in NAFLD patients compared to control groups [61,110]. The liver-specific miR-122, which affects cholesterol biosynthesis* in vivo*, has been shown to promote adipocyte differentiation [114]. Recent animal studies have found that the genetic deletion of miR-122 in mice resulted in hepatic steatosis, inflammation, and HCC [62,115]. In a mouse model, the plasma cholesterol level, hepatic fatty-acid and cholesterol synthesis rate, and the level of hydroxy-methyl-glutaryl coenzyme A reductase (HMGCR) that produces cholesterol, were also significantly decreased after repression of miR-122 [114]. These findings strongly suggest that the miR-122 is an important regulator of lipid metabolism in the liver and consequently the miR-122 acts as a tumor-suppressor in the liver [8].

Besides miR-122, some other miRs have been shown to be involved in NAFLD development. miR-21, miR-23a, miR-34a, miR-143 and miR-146b were demonstrated to be significantly over-expressed in human NAFLD and NASH [8,61]. The enhanced miR-335 expression was also associated with increased white adipose tissue weight, as well as elevated hepatic TG levels. Furthermore, hepatic miR-335 level closely correlated with the expression of adipocyte differentiation markers including PPAR-α [63]. Interestingly, emerging evidence indicates that miR-21, miR-103, miR-143 and miR-378 increase oxidative stress and inflammation in animal models with obesity and steatosis [108].

Recently, new research suggests that folate status may influence the miR expression linked to the severity of fatty liver disease [49]. Folate supplementation seems to affect the expression of miRs possibly through changes in methylation levels of promoter regions in the corresponding miR gene [116]. The severity of NAFLD induced by folate-deficient diet in mice is associated with the altered expression of hepatic miRs, including miR-181a, miR-34a, miR-200b, and miR-221 [117]. Furthermore, some of the differentially-expressed miRs, which function in hepatic lipid and glucose metabolisms, have been shown to promote the development of HCC in NAFLD [8].

In hepatocytes, unsaturated fatty acids trigger steatosis by inducing NF-κB signaling and concomitantly activating miR-21, which, in turn, directly suppress the expression of the phosphatase and tensin homolog gene (*PTEN*) [118]. Inhibition of miR-21 increased *PTEN* expression and decreased HCC tumor cell proliferation and migration, suggesting that the miR-21 and tumor suppressor *PTEN* pathway are involved in the NAFLD-related HCC development [119]. Additionally, miR-23a up-regulation was also observed in both human NASH and HCC, and the activation of the IL-6/STAT3/miR-23a pathway could promote hepatocarcinogenesis via the alteration of the glucose homeostasis [120]. Hepatic miR-155 was also found to be over-expressed in diet-induced NASH models, and the up-regulation of miR-155 was associated with the early stages of hepatocarcinogenesis [64,121]. Moreover, NF-κB activates miR-155 expression, thereby linking NF-κB signaling to NAFLD-related HCC via miR deregulation [122].

## 4. Nutritional Intervention through One-Carbon Metabolism

**Figure 1:**
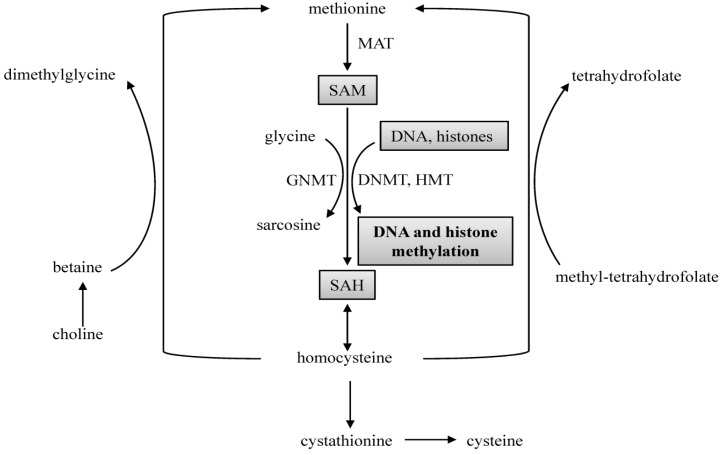
One-carbon metabolism.

SAM, *S*-adenosylmethionine; SAH, *S*-adenosylhomocysteine; DNMT: DNA methyltransferase; HMT: histone methyltransferase; MAT: methionine adenosyltransferase; GNMT: glycine *N*-methyltransferase.

Liver is central for one-carbon metabolism, which is a biochemical network that delivers methyl group (one-carbon moiety) to the biological methylation pathway and nucleotide synthesis pathway. In one-carbon metabolism, water soluble B vitamins, including folate, vitamin B12, B6 and B2, act as coenzymes. Sulfur containing amino acids such as methionine, homocysteine and cysteine are involved in the synthesis of *S-*adenosylmethionine (SAM), and choline and betaine are folate independent source of methyl groups for homocysteine remethylation (Figure 1). Dysregulation of one-carbon metabolism, especially SAM biosynthesis, may alter the hepatocyte function, leading to fatty liver disease [123]. SAM is the unique methyl donor for many biological methylation reactions including the methylation of DNA and histone [84]. After transferring the methyl group, SAM is converted to *S-*adenosylhomocysteine (SAH), which is an inhibitor of methyltransferases such as DNMTs and HMTs [124]. Recent studies have demonstrated that imbalance of SAM and SAH by over-nutrition or under-nutrition can trigger epigenetic changes, thereby linking nutrients to epigenetic gene regulation in cell proliferation and survival [75,125,126].

Indeed, alterations in hepatic SAM and folate status were observed in obesity and metabolic syndrome that have hepatic steatosis [49]. Mouse models with genetic ablation of the methionine adenosyltransferase 1A gene (*Mat1a*), which encodes the enzyme that catalyzes the conversion of methionine to SAM, showed lower hepatic SAM level, higher lipogenesis and oxidative stress, and consequently were predisposed to the NAFLD-related HCC development [65,66]. Interestingly mice deficient in the SAM catabolic enzyme, glycine *N-*methyltransferase (*Gnmt*) that showed extremely high hepatic SAM levels also developed steatohepatitis and HCC spontaneously [67]. Thus, the maintenance of adequate hepatic SAM levels is critical for hepatic energy metabolism and lipid homeostasis [8,127]. Either too much or too little SAM in the liver may cause aberrant DNA methylation and epigenetic dysregulation of metabolic pathways [128].

A body of evidence has shown the comprehensive association between folate, choline, and hepatic lipid metabolism [129,130]. An important role of folate is in the maintenance of hepatic SAM and SAH balance [49]. Folate deficiency reduces *de novo* phosphatidylcholine (PC) synthesis resulting in accumulation of hepatic TG and NAFLD [131]. In murine models, changes in PC synthesis affected hepatic lipid storage and secretion, then it was predisposed to the development of steatosis [132,68]. Impaired PC production is associated with accumulation of hepatic TG due to a reduction in VLDL secretion [68]. Low dietary folate is associated with hepatic steatosis but it does not necessarily indicate that folic acid supplementation may prevent fatty liver disease [49].

In humans, folic acid, an oxidized synthetic form of folate, can effectively turn to the active form of folate, methyltetrahydrofolate, at low levels of intake up to 100 µg folic acid per day [117]. Intake above 200 µg/day, however, is the threshold at which folic acid begins to appear in the blood stream in the un-metabolized form, which may inhibit remethylation of homocysteine [133]. Thus, the notion that high intake of folic acid over the threshold may rather disturb one-carbon metabolism has been postulated [134,135]. On the other hand, supplementing the maternal diet with folate and choline increased the DNA methylation status of the *agouti* gene and subsequently reduced the development of obesity and NAFLD in the *agouti* mouse model [3].

A most recent study revealed that the supplementation with l-carnitine, which requires SAM-dependent methylation for its synthesis, showed improvements in both clinical and histological aspects of liver damage in NASH patients [69]. Nevertheless, it is unclear whether fatty liver can be directly attributed by the reduction of carnitine synthesis due to the impaired methylation capacity [49]. Future studies examining the potential role of carnitine in the development of steatohepatitis are needed.

## 5. Epigenetic Intervention Using Dietary Natural Compounds

Recently, the beneficial effects of dietary natural compounds in the prevention and treatment of NAFLD have been reported [136]. Many natural compounds, especially polyphenols, isolated from fruits and vegetables have health promoting properties of anti-inflammation, anti-oxidation and anti-obesity. They also show hepatoprotective effects mainly by reducing lipogenesis and increasing the fatty acid oxidation in hepatic lipid metabolism [137]. Some of their chemopreventive functions are related to epigenetic modulation in some part, which have been demonstrated in animal and clinical studies.

Resveratrol, a compound found largely in the skins of red grapes, is widely accepted as a chemopreventive agent and exerts its health benefits via anti-oxidative, anti-inflammatory, anti-cancer and anti-diabetic properties. Recent studies showed that resveratrol attenuates palmitate-induced deregulation of insulin signaling and endoplasmic reticulum stress through the activation of SIRT1-induced oxygen-regulated protein 150 [138]. Alberdi* et al.* reported that supplementation with low dose resveratrol in high-fat diet fed mice protected hepatic steatosis, via increased activities of palmitoyl transferase-1A and acyl-CoA oxidase, two enzymes involved in fatty acid oxidation [139]. This protective action of resveratrol was, in part, mediated through the up-regulation of SIRT1-AMPK signaling system.

Other polyphenols, such as antocyanin Cy-3-g, proanthocyanidins, teaflavin and ellagic acid (a tannin), have been studied as potential agents for both prevention and treatment of NAFLD [137]. Baselga-Escudero* et al.* conducted a study of mice fed with proanthocyanidins from grape seed dissolved in the lard oil [140]. After supplementation of proanthocyanidins, a reduction in hepatic TG content was observed with increased miR-122 mRNA levels accompanied by decreased Fas cell surface death receptor (*Fas*) mRNA levels. Aoun* et al.* reported the supplementation effect of Provinol^®^, a polyphenol extract obtained from red wine, in a high fat diet animal model [141]. A reduction in liver TG accumulation and hepatic lipid peroxidation were observed and the activation of Sirt1 has been suggested as a potential mechanism of these effects.

Until now, these beneficial effects of natural compounds have been demonstrated mostly in animal models. Thus, human studies are needed to confirm the real efficacy of natural compounds in the prevention and treatment of NAFLD patients.

## 6. Conclusions and Future Perspectives 

It is quite clear that the field of nutritional epigenetics is further clarifying the mechanisms of gene-nutrient interaction, providing the role of nutrition in determining phenotype from genotype [84]. Thus, there is great interest in identifying epigenetic-based therapeutic strategies as a means to prevent the development of NAFLD related conditions. Even though knowledge regarding the effects of epigenetics on NAFLD is limited, epigenetic intervention is becoming a new and rapidly growing field for potential therapeutic strategies aimed at preventing diseases by reversing the epigenetic aberrancies. Epigenetic therapeutics could be directly targeted to epigenetic modifying enzymes [45,142] or indirectly targeted to one-carbon metabolism that controls the methylation of DNA and histones. In addition to potent epigenetic cancer chemotherapeutic agents, many botanicals have been identified as possible epigenetic modulators that ameliorate the metabolic syndrome and NAFLD [37,143]. To date, many reports have indicated that phytochemicals including epigallocatechingallate (EGCG), resveratrol, genistein, curcumin, and isothiocyanates can modify the enzymatic activities of DNMTs, Class I, II, IV HDACs, Class III HDAC SIRT1, and HATs, beneficially modulating inflammatory responses in metabolic syndrome [3,144]. As described above, there are extensive alterations in miRs in NAFLD, and the modulation of those critical miRs expression could also be an effective approach to NAFLD and NASH patients.

The HCC is highly heterogenic from the perspectives of genetic and epigenetic mechanisms. The investigations underlying epigenomic aberrations that involve in the malignant transformation of hepatocytes could lead to the development of preventive and therapeutic strategies for NASH-associated HCC [8,145,146]. Through the studies regarding the relationship between the metabolic dysfunction and chromatin dynamics in NAFLD and HCC, eventually we could determine how metabolic syndrome reprograms the epigenome in the liver and whether the altered epigenetic patterns can be reversed [147,148]. Nowadays, new players of chromatin remodeling proteins and histone modifying proteins in the progression of NAFLD are being identified [99,149,150]. Furthermore, Genome Wide Association Studies that identified original gene-disease interactions is now being applied to functional epigenetics, which can guide us to the new arena of Epigenome-Wide Association Studies of NAFLD and associated morbidity [84].

The changes in DNA methylation, histone modifications as well as altered expression of miRs, all together can promote the development of NAFLD by altering lipid and glucose metabolism. Those epigenetic modifications claim a large proportion of disease phenotypes. Further research is needed to understand the basic epigenetic mechanisms by which diverse nutrients modify the development and progression of NAFLD for the ultimate purpose of avoiding the serious complications.
